# Modeling and scale-bridging using machine learning: nanoconfinement effects in porous media

**DOI:** 10.1038/s41598-020-69661-0

**Published:** 2020-08-07

**Authors:** Nicholas Lubbers, Animesh Agarwal, Yu Chen, Soyoun Son, Mohamed Mehana, Qinjun Kang, Satish Karra, Christoph Junghans, Timothy C. Germann, Hari S. Viswanathan

**Affiliations:** 1 Information Sciences Group, Computer, Computational and Statistical Sciences Division, Los Alamos National Laboratory, Los Alamos, NM 87545 USA; 2 Theoretical Biology and Biophysics Group, Theoretical Division, Los Alamos National Laboratory, Los Alamos, NM 87545 USA; 3 Computational Earth Science Group, Earth and Environmental Sciences Division, Los Alamos National Laboratory, Los Alamos, NM 87545 USA; 4 Institut des Sciences de la Terre, Université Grenoble Alpes, Grenoble, France; 5 Geophysics Group, Earth and Environmental Sciences Division, Los Alamos National Laboratory, Los Alamos, NM 87545 USA; 6 Applied Computer Science Group, Computer, Computational and Statistical Sciences Division, Los Alamos National Laboratory, Los Alamos, NM 87545 USA; 7 Physics and Chemistry of Materials Group, Theoretical Division, Los Alamos National Laboratory, Los Alamos, NM 87545 USA

**Keywords:** Crude oil, Natural gas, Computational science, Fluid dynamics, Computational nanotechnology

## Abstract

Fine-scale models that represent first-principles physics are challenging to represent at larger scales of interest in many application areas. In nanoporous media such as tight-shale formations, where the typical pore size is less than 50 nm, confinement effects play a significant role in how fluids behave. At these scales, fluids are under confinement, affecting key properties such as density, viscosity, adsorption, etc. Pore-scale Lattice Boltzmann Methods (LBM) can simulate flow in complex pore structures relevant to predicting hydrocarbon production, but must be corrected to account for confinement effects. Molecular dynamics (MD) can model confinement effects but is computationally expensive in comparison. The hurdle to bridging MD with LBM is the computational expense of MD simulations needed to perform this correction. Here, we build a Machine Learning (ML) surrogate model that captures adsorption effects across a wide range of parameter space and bridges the MD and LBM scales using a relatively small number of MD calculations. The model computes upscaled adsorption parameters across varying density, temperature, and pore width. The ML model is 7 orders of magnitude faster than brute force MD. This workflow is agnostic to the physical system and could be generalized to further scale-bridging applications.

## Introduction

Multi-scale physics problems are found in all scientific disciplines. Prominent examples can be found in material science^1–3^, biology^4^, chemistry ^5–9^, and geosciences^10–12^. Typically, information from computationally intensive fine-scale models have to be translated or upscaled into faster coarse-scale models to solve the problem at the scale of interest. A problem of great scientific and economic interest is the flow of hydrocarbon in nanoporous shale. Traditional porous media approaches such as the LBM allow for complex pore geometries but need to be provided with effective properties that account for nanoconfinement effects in order to accurately simulate mass transport at the continuum scale^13^. Atomistic simulations such as Molecular Dynamics (MD) capture nanoconfinement effects accurately, but are limited to a few pores as they are computationally intractable to simulate for mesoscopic pore geometries. There is a need for approaches that efficiently bridge these two scales without compromising accuracy.

Recently, Machine Learning (ML) has shown great promise in accelerating physics-based models that makes it feasible to build a scale-bridging framework^14–16^. The applications include fracture propagation in brittle materials^17^, computational fluid dynamics^18^ and molecular dynamics^19^. On another dimension, Machine Learning (ML) techniques have found their way into petroleum engineering and studies of porous media. For instance, Fulford et al. ^20^ used ML to tackle the challenges for predicting well performance in shale reservoirs, and similarly, Li et al. ^21^ used an ensemble of ML techniques to construct the expensive-to-acquire logs which provided a reliable way to estimate the in-situ geomechanical properties of shale reservoirs. Kamrava et al. ^22^ used ML to generate synthetic 3D micropore structures in shale. Additionally, Kamrava et al. ^23^ estimated permeability of structures. Combining both accelerated computation and porous media, Santos et al. ^24^ modeled complex fluid flow through 3D porous media geometries. In this work, we describe and implement an ML framework to bridge the molecular and continuum scales in order to accurately simulate hydrocarbon adsorption in nanoporous media.

The physics of nanoconfined hydrocarbons has come to the forefront due to the recent unconventional boom. Hydrocarbon-rich tight formations (low-permeable shale, sandstone, and carbonate rock formations) have very small pores ranging in size from a few to a few hundred nanometers. Hydrocarbons are either stored as free oil/gas in the pore space or adsorbed on the pore walls. Because of the small pores and the resulting low permeability ($10^{-16}$ to $10^{-20}\ \hbox {m}^2$)^25^, the enclosed hydrocarbon resources are very difficult to access. Hydraulic fracturing and horizontal drilling allow access to the free oil/gas in the fracture network and adjacent damaged zones but the hydrocarbons in the matrix are still untapped^26, 27^. If the mass transport from the matrix can be accelerated and later-stage production can therefore be improved, then the petroleum industry may find it profitable to continue production from existing wells before drilling new ones, leading to enhanced recovery while minimizing the environmental impact.

However, since hydrocarbon in shale matrix is under nanoconfinement, due to the small pore sizes (e.g., less than 50 nm), it prevents traditional reservoir simulators from accurately predicting mass transport from the shale matrix into the fractures. Specifically, properties such as density, viscosity, phase transition, and adsorption deviate from macroscopic behavior under nanoconfinement due to the increased importance of boundary layer effects, greatly affecting mass transport rates^28–39^.

Adsorption, the accumulation of hydrocarbon molecules onto the pore walls, is one of the most critical nanoconfinement processes that affects the extraction of hydrocarbons out of nanopores^40^. It is estimated that a large portion (20–80%) of the total shale gas in a reservoir is in the absorbed form^40, 41^. Methane adsorption under nanoconfinement and its effect on transport in shale matrix have been investigated through off-line MD and LBM^35^. In that study, equilibrium MD simulations are conducted to study methane adsorption on the organic and inorganic walls of nanopores in shale matrix with different pore sizes and pressures. Density and pressure distributions within the adsorbed layer, as well as the pressure-dependent thickness of adsorbed layer, are obtained from the MD simulations. This information is then implemented in the LBM simulations, through which the effect of adsorption on transport is considered. However, in this approach, it is implicitly assumed that there is a clear separation of scales. This is not a generally valid assumption, since confinement effects in the pore affect flow through the nanoporous medium and vice-versa.

We demonstrate our ML-based scale-bridging framework to capture adsorption under nanoconfinement where there is no clear separation of scales. We incorporate atomistic adsorption effects that occur within a nanoconfined pore as simulated accurately by MD into a continuum LBM that is capable of simulating larger scales. We show that our ML framework is accurate and much more efficient than direct MD, allowing up to 7 orders of magnitude speedup making it ideal for a robust scale-bridging framework. Moreover, the workflow is not dependent on the physical characteristics of adsorption phenomena; the workflow is agnostic to the physical system at hand and could be generalized to further surrogate modeling and scale-bridging applications.

## Results

**Figure 1:**
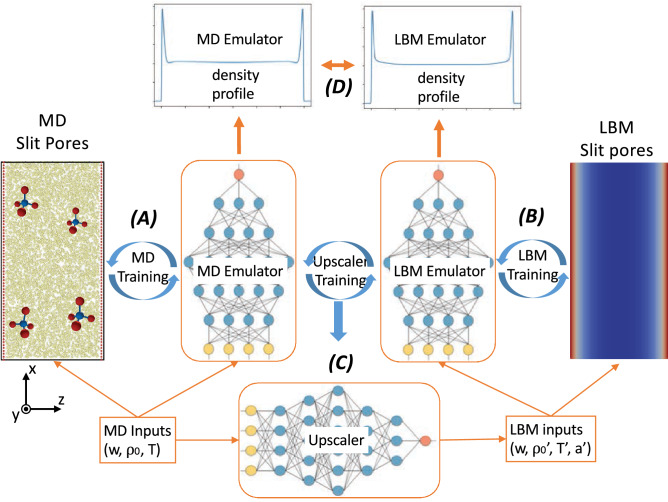
Our machine learning based scale-bridging framework. DNN emulators are constructed for both fine (MD) scale processes (**A**) and coarse (LBM) scale processes (**B**) by modeling the entire pore profile based on datasets which span a range of pore conditions.The differentiability of the emulators is exploited to train the DNN upscaler (**C**) by training it to match the density profiles between MD and LBM profiles (**D**) across the parameter domain. The resulting upscaler finds the effective parameters (in this case effective density, effective temperature and adsorption parameter) for the coarse scale model informed by the fine scale data.

In order to bridge the scales between MD and LBM, we take advantage of the recent advances in ML, where Neural Network based emulators can be used to replace physics-based models. Our goal is for LBM to accurately model adsorption under nanoconfinement, requiring that it be informed by MD. To achieve this, we aim at building an upscaler that maps MD inputs to LBM inputs. Both models require pore width, overall density and temperature as inputs to capture adsorption behavior. However, since LBM is a continuum model, an additional adsorption coefficient is needed to capture the fluid-wall interaction in the LBM method. This parameter cannot be directly measured, rather, it is a model parameter that must be calibrated. Since both MD and LBM can be computationally expensive to span the entire input parameter space, in order to build the upscaler, first we build emulators that mimic MD and LBM behavior. Specifically, we utilize Deep Neural Networks (DNNs) as our emulators. We train the emulators using apriori MD and LBM simulations over their respective input parameter spaces (Fig. 1A,B). Our emulators also ensure conservation of mass to prevent unphysical density profiles, and ensure that our emulators produce smooth, symmetric profiles. This is done through engineering the architecture of the NN via constraining the activation functions of the neurons. The spatial density profile is used as the output for both MD and LBM and is representative of the adsorption of the fluid to the pore wall. Having trained two emulators, we train a DNN-based upscaler (Fig. 1C) that maps MD inputs into LBM inputs. The goal of the upscaler is to match the spatial density profiles between the MD and LBM emulators. This is done by finding the optimal density, temperature and the adsorption coefficient that minimize the error between the spatial density profiles from the two emulators (Fig. 1D).

The choice of DNN models is an important one, motivated by the availability of fast Automatic Differentiation algorithms for computing gradients ^42^ that are already implemented in DNN libraries ^43^. Training the upscaler requires gradient information about changes in LBM profiles with respect to LBM inputs. These gradients are easy to compute from the DNN-LBM emulator, and allow us to use the emulated profile behavior to train an upscaler with less computational effort and human time than would be necessary otherwise. In contrast, it would be far more expensive to calculate numerical finite-difference gradients from discrete LBM simulations, which requires many LBM simulations for any given state point to determine the sensitivity of the profile with respect to the input parameters. Similarly, using automatic gradients is far less laborious than deriving, coding, and testing the gradient of LBM profiles with respect to LBM input combinations within an existing LBM code. In addition, the LBM emulator is about 4 orders of magnitude cheaper than direct LBM (see “Computational costs”), making it more tractable for training the upscaler.

Next we summarize the gaps with traditional scale-bridging methods and the advantages to our approach:

1. Sequentially approaching adsorption one input parameter at a time will require a separate set of MD calculations for each parameter to consider, which is computationally laborious. Ours is a holistic method over the entire multivariable parameter space and requires few MD simulations to perform upscaling.
2. Because the framework operates over a multivariable parameter space rather than optimizing upscaled parameters on a point-by-point basis, it is suited for the simultaneous modeling of multiple upscaled parameters, because it incorporates the properties of many simulations simultaneously.
3. Upscaled functions are often assumed to obey some sort of relationship such as a linear or exponential dependence in order to simplify the procedure. Our approach makes no assumptions about the form of upscaling functions from fine-scale (MD) inputs to coarse-scale (LBM) parameters, except that these functions can be generated by a neural network.
4. Our framework is extensible to other forms of upscaling such as flow conditions, nanoscale modifications to equation of state, and phase transition characteristics.
5. In this work we focus on 1D pores; however, the overall workflow does not depend on the dimensionality of the system, and can be generalized to two and three dimensional porous geometries.
6. The method enables extreme throughput for upscaled parameters. Once trained, calls to the upscaler are very cheap, and can easily be evaluated millions of times at negligible computational cost. This is highly advantageous in comparison to direct scale-bridging methods, which require continual calls to fine-scale simulations to advance the coarse-scale simulation.

In the 1D pore scenario studied here, the inputs to an MD simulation are width, overall methane density, and temperature, denoted *w*, $\rho _o$, *T*. LBM simulations use the same width *w*, and take density $\rho _0'$ and temperature $T'$, as well as the additional adsorption parameter, denoted $a'$. The role of the upscaler is to take any set of inputs *w*, $\rho _0$, and *T*, and determine matching effective parameters $\rho _0'$, $T'$, and $a'$ such that adsorption effects in the LBM physics match those in MD.

**Figure 2:**
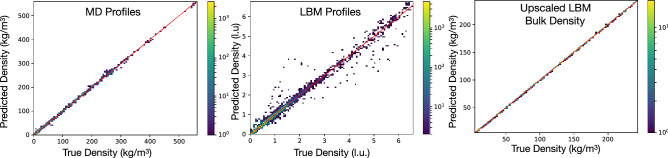
Left: MD emulator performance for all profile points in the test dataset, Middle: LBM emulator performance for all profile points in the test dataset. Right: Upscaler performance for bulk density in the test dataset. Each plot is a two dimensional histogram; the corresponding color bar indicates the number of points in each bin.

We use datasets of 1,010 simulations for MD and 8,074 for LBM (for details, see “Methods” below). The parameter ranges for MD are: 3–300 kg/m^3^ for density, 300–400 K for temperature and 2.4–22 nm for pore width. For LBM, the parameter ranges are (in lattice units, l.u.): 0.25–1.5 l.u. for density, 0.8–2 l.u. for temperature, 3–13 nm for pore width, and 1–10 for the (dimensionless) adsorption parameter. The MD and LBM emulators were then trained on these simulations. The training for the MD and LBM emulators shows good overall profile density, and the upscaler network produces good models of the bulk density, as shown in Fig. 2, which compares the true and predicted values on held-out test fractions of the datasets. We note that the MD plot compares the collected (True) and emulated (Predicted) profiles across each point and shows very little scatter. The LBM plots the same quantity for LBM simulations. While there are a few outlier points, the vast majority of the predicted points fall very close to the to true points. The upscaler is trained to construct LBM inputs that generate the correct bulk density, and so the resulting plot only has one point per profile. For the upscaler, the fit visually near perfect.

### Extracted physical quantities

**Figure 3:**
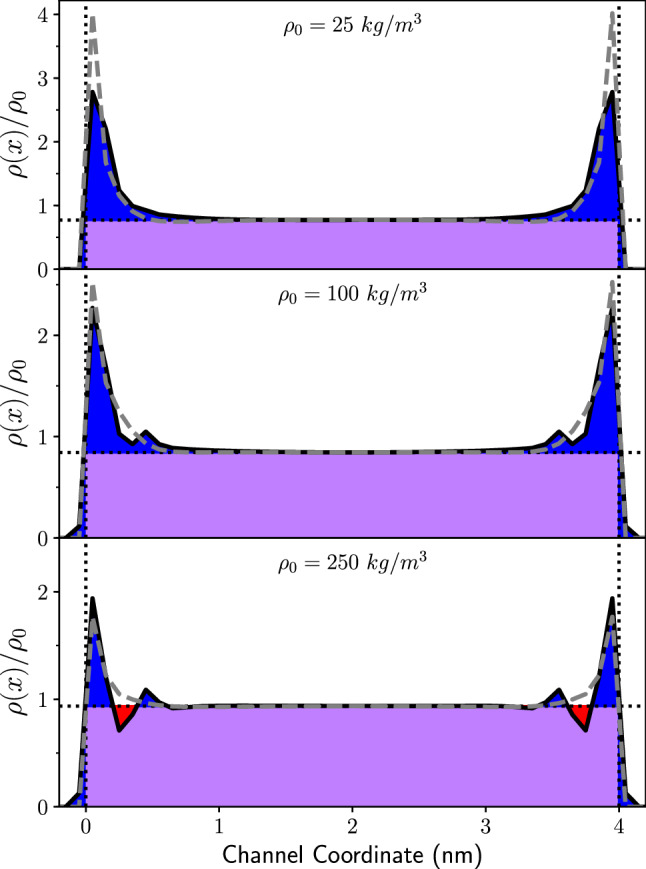
Emulated and upscaled profiles for $T=350 K$ and pore width $w=4~\hbox {nm}$. From top to bottom, panels show input densities of $\rho _0 = 25, 100, 250$ kg/m^3^, respectively. The black curve shows the emulated MD profile, and the dashed grey curve shows the corresponding upscaled profile. Colored regions show how to compute the MD excess density compared to an adsorption-free fluid with the same bulk density: the purple region shows the coincidence of the MD profile with the adsorption-free fluid, the blue region shows excesses compared to the adsorption-free fluid, and the red regions show deficits compared to the adsorption-free fluid.

The trained emulators and upscaler can be used to extract physical information about the pore. Figure 3 shows our approach in comparing profiles to a uniform profile with the same bulk density, where bulk density is defined as the density in the center of the pore. Blue regions show excess density compared to the bulk. The excess density represents the nanoconfinement adsorption effect from MD that needs to be captured in LBM through the adsorption parameter. The excess density is calculated as follows. The purple region shows the intersection of the MD profile with the adsorption-free fluid profile, the blue regions are the excess compared to the adsorption-free fluid profile, and the red regions are deficits compared to the bulk (central) density. The excess density is then given as the total area of the MD profile (purple plus blue) minus the total area of the bulk profile (purple plus red). Interestingly, as the methane density increases, the width of the emulated adsorption layer decreases. Although multiple adsorption layers form in MD, as the density is increased further, a deficit forms (red region in Fig. 3) between the first and second layers, which, overall, mitigates the excess density in the second adsorption layer. This signals an increase of structure in the adsorbed particles. This phenomenon naturally arises from the microscopic nature of MD which is captured in our workflow without apriori conceptualization.

**Figure 4:**
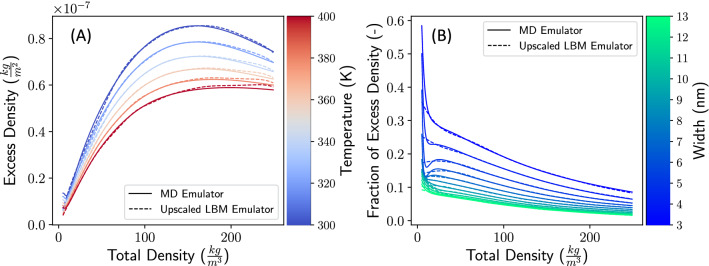
Excess density for a variety of pore conditions. Solid lines show the results of the MD emulator, and dashed lines show the results of the Upscaled LBM emulator. Left: Excess density as a function of temperature and total density $\rho _0$ for a fixed width of 4 nm for pore, for temperatures ranging from $T=300$ K to $T=400$ K. Right: Fractional excess density as a function of pore width and total density $\rho _0$ at $T=350$ K, for widths ranging from $w=3$ nm to $w=13$ nm.

Figure 4 compares the excess density across the input space, and indicates that measurements from the MD and upscaled LBM emulators are in good agreement. As seen in Fig. 4A, for low densities, the excess density is approximately linear with the total density. When the density is near 150 kg/m^3^, a turning point is approached, and excess density begins to decrease with increasing total density. This corresponds to a saturation of the adsorbed particles near the wall; any new particle added to the system is more likely to equilibrate into the bulk than to the adsorbed layer. At extremely low densities below $\approx 5$ kg/m^3^, there is a small mismatch between the MD emulator and the LBM upscaler—we will remark on this further at the end of this section. The multiple curves for various temperatures indicate that this effect is present for all temperatures, but the saturation effect occurs at larger densities for higher temperatures. Figure 4A also shows that excess density is inversely related to temperature. This demonstrates that as temperature increases, methane preferentially desorbs from the wall into the bulk. This effect is captured in the upscaling scheme.

Figure 4B shows the fraction of excess methane density compared to total density. We found that the nano-confinement allows the nanopores to pack more methane compared to the bulk-based estimates. Quantitatively, one can expect at least 15% more methane in 4 nm pores compared to the bulk-based estimates, which is consistent with prior suggestions^44^. This better packing is more pronounced at lower densities and smaller pores. Note that what we report as the fraction of the excess density is different than the fraction of the adsorbed phase. The excess density reports the ratio of the excess mass, the total mass minus the uniform bulk mass, to the total mass, a quantity which can easily be obtained from an MD profile. The total adsorbed density represents the ratio between the mass of the adsorbed phase to the total mass in the pore. Performing such a measurement requires separating the methane density into separate adsorbed and bulk phase components, and such a distinction cannot be physically made from an MD density profile. Because of the difference in the nature of these two quantities, our reports of excess density differ significantly from the total adsorbed mass reported in the literature ^45^. We note that for extremely low total densities below $\approx 5$ kg/m^3^, the fraction of excess density exhibits observable disagreement between MD and LBM emulators.

**Figure 5:**
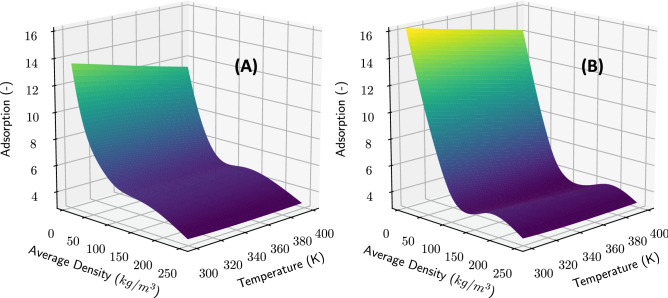
Upscaled LBM adsorption coefficient as a function of temperature and density for a fixed pore-width of 4 nm (**A**) and 12 nm (**B**). Each 3D surface is colored by the value of the the adsorption coefficient.

Figure 5 shows the adsorption coefficient predicted by the upscaler, that is used by LBM to model adsorption under confinement, for a wide range of temperatures and total densities, at pore widths of 4 nm and 12 nm. As seen in the figure, the relationship between the adsorption coefficient, total density, and temperature is smooth but complex. This demonstrates that scale-bridging is necessary for informing the LBM adsorption coefficient under confinement. The adsorption coefficient is strongly increasing as the total density decreases, but varies less for large densities. The adsorption coefficient shows a weaker monotonically increasing dependence on temperature. For the 4 nm pore the density dependence is monotonic—this may be a region where nano-confinement induces a supercritical phase^46^. On the other hand, in a 12 nm pore, the non-monotonic trend of adsorption with density may be indicative of a sub-critical phase. We reiterate that this functional form for the LBM adsorption coefficient was not selected a priori, but was generated by training the upscaler through the fusion of information from MD and LBM simulations.

Finally, we discuss the performance of the MD and upscaled LBM emulators for very low density systems (below $\approx 5$ kg/m^3^), where there is some disagreement between the two systems. This discrepancy is small in absolute terms (Fig. 4A) and noticeable in relative terms (Fig. 4B). There are several possible sources of this discrepancy, each with accompanying remedies:

1. The mismatch may stem from the differences between MD and LBM formulations. The discrete nature of MD allows the accumulations of molecules near the surface with no presence of molecules in the center of the pore, that is, the system consistents entirely of adsorbed particles, with essentially zero in the bulk. On the other hand, in LBM simulations, there must be a continuum between the bulk fluid and the the adsorbed fluid; the LBM formulation may not be able to treat such low densities. If this is the case, upscaling cannot be performed for extremely low densities simply because the physics of the fine scale cannot be represented at the coarse scale.
2. It may arise because the cost function for the upscaler has units of density (see “Methods”). This explains why the mismatch is not large in absolute terms but magnified in relative terms. The solution in this case is to specify the cost function in terms of the physical observables of interest; if the variable of interest is fractional pore adsorption, then the accompanying cost function should be dimensionless.
3. It may be due to the large adsorption coefficients computed by the upscaler for low densities. The LBM dataset was limited to adsorption coefficients less than 10, but for low densities the upscaler drives the emulator out of this range (Fig. 5). One might address this case by extending the range of LBM simulations to larger adsorption values.

We expect that some mixture of these effects accounts for the situation. It is worth noting that the common reservoir conditions in shale formations are unlikely to probe such low densities; as such we expect it to be largely irrelevant to applications. Despite this, we present this discussion as it entails several considerations for future research into ML-based upscaling: One is to keep in mind that it is important to consider the range of physics accessible at the coarse scale. Second, one must carefully consider which properties are intended to be probed and how they give rise to a natural cost function to optimize; small mismatches in one metric may imply larger mismatches under another metric. The last is to consider the range of data sampled: While ML is designed to generalize to new data that is similar to training data, no purely data-driven method could be expected to extrapolate to conditions far outside of the range of data sampled.

### Computational costs

The aim of scale-bridging techniques is to alleviate computational costs associated with brute-force fine-scale simulation while retaining the important aspects of the fine-scale physics, and so in this section we review the computational cost of our methods. MD and LBM calculations were performed on various commericially available HPC hardware with CPU-based implementations. Neural networks were treated with a 12-core Intel i9 CPU on a commercially available laptop.

First, we cover the data-collection and model-building phases. Collecting the 1,010 MD simulations required approximately 800 core-h of computation ($\approx$ 1 core-h/MD-call). It is worth noting that this is comparable to the number of fine-scale calls needed in a single time-step in scale-bridging applications ^1–3, 6^. The matching 8,074 LBM simulations are far simpler, taking $\approx$20 core-h total, or $\approx$5 core-s/LBM-call. Training the MD emulator, LBM emulator, and upscaler took approximately 24 core-h each, resulting in $\approx$72 core-h of training time. The dominant cost is thus MD simulation.

Having trained our models, the neural networks can be evaluated very quickly. The emulator must treat the density across the entire pore, and on average takes $\approx$1 core-ms/emulator-call. We remark that generating Fig. 4 requires assessing the profile of $\approx 3200$ sets of densities and temperatures for a fixed-width pore; this is considerably larger than the number of MD calculations performed in total over all pores. The emulators can generate this data in seconds on a single machine. The upscaler does not need to be evaluated for each point in the profile, and so it can be evaluated in $\approx$200 core-$\upmu$s/upscaler-call in serial evaluation. If batched to a sufficient number of combined calls, this can be lowered to $\approx$10 core-$\upmu$s/upscaler-call. If we take the serial mode as a conservative estimate, calling to the upscaler is $10^7$ times cheaper than a single MD simulation.

There are many practical considerations necessary to contexturalize the $10^7$ speedup factor, which can be regarded as an asymptotic speedup possible when other effects do not dominate. First, we mention potential limitations. One is that acheiving this speedup factor requires an application where the number of upscaling calls required is $10^7$ times larger than the number of training MD simulations. While this is large, current supercomputers exceed $10^5$ total cores ^47, 48^, and atomistic applications have utilized computation at this scale ^49–52^. Another factor is the expense of coarse-scale operations; the speedup factor of our method compared to direct MD is limited by the ratio of computation spent in the coarse model compared to MD. Lastly, implementation details may give rise to overheads associated with communication of data, etc. that affect final performance.

We also consider the practical advantages of the workflow. For one, the training MD simulations that constitute the bulk of the cost can be massively parallelized to take advantage of large HPC environments; in direct scale-bridging, parallelization is limited to the number of MD calls needed in a given time-step. Second, with such a large asymptotic speedup factor, larger coarse-scale simulations can be designed to take advantage of the throughput available with the ML-based upscaler. Third, given that 800 core-h for MD with a united-atom force field can be performed on a small cluster, far more accurate potentials could be afforded. In this case, the speedup factor would be proportionately larger; changing the nature of the MD simulation will not affect the cost of ML. Lastly, all three core algorithms (MD, LBM, and DNN) are good applications to accelerate via GPGPU computation ^53^.

## Conclusions and future prospects

In this work, we have described and demonstrated an ML framework to bridge the molecular and continuum scales in order to accurately simulate hydrocarbon properties in nanoporous media. We incorporate atomistic adsorption effects that occur within a nanoconfined pore as simulated by MD into a continuum LBM that can simulate larger scales with more complex geoemetries. We demonstrated that our ML framework is accurate and much more efficient than direct MD allowing up to 7 orders of magnitude speedup making it ideal for a robust scale-bridging framework that could be deployed even on modest computer clusters. Our approach is not dependent on the physical characteristics of adsorption phenomena. Therefore we expect to be able to extend it to other nanoconfinement effects such as slip and phase transition effects that also affect hydrocarbon flow, but have thus far been neglected in traditional continuum reservoir simulators. Our framework is flexible and extensible to other forms of upscaling. In addition, we expect the flexibility of the upscaling form to provide additional physical insights into how discrete molecular effects manifest themselves in complex 3D pore structures at the continuum scale. If an unexpected upscaling function emerges during our analysis, it could illuminate new physics within a nanoconfined pore. For example, as methane density increases, the adsorption thickness actually increases due to the formation of multiple adsorption layers. This was not explicitly parameterized in our framework but was discovered in the data analyis.

The advantages of our approach were demonstrated for an adsorption application. Our method simultaneously captures adsorption for a wide range of temperatures, pore widths and densities. Prior approaches require re-fitting of upscaling parameter; for example, a change in temperature or pore width implied the need to re-establish the adsorption as a function of total density. In contrast, the ML approach captures the data in one-shot across the full parameter space. While prior approaches modeled adsorption purely as a relationship between bulk and total density, our approach models the entire density profile and calculates the optimal adsorption parameter in the upscaler. This enables validation by ensuring that the estimated profiles are physically realistic. We also constructed our emulators such that they ensure symmetry and conservation of mass to prevent unphysical density profiles. This is done through engineering the architecture of the emulators via engineering the activation functions of the neurons. DNNs prove useful in this regard because their engineerable architectures lend themselves to applying physics informed constraints to the model.

Analyzing the resulting models shows that it captures many physical effects present in MD by dynamically adjusting the parameters of LBM. The performance degrades slightly near the edge of the input space when density becomes very low. Computationally, calls to the trained upscaler are essentially free, and the dominant cost is in producing MD training data. While the cost is not large, it is dominant in the workflow, and would increase when studying systems that require more complex potentials, such as biomolecules or metals with long-range forces. Minimizing the computational cost of acquring data is a good candidate for future work. It is a complex endeavor, involving both continuum and molecular simulation methods, model hyperparameters, and the choices of learning objective. One method to tackle challenge would be Active Learning ^54^, in which an algorithm is applied to automatically select new training data in order to improve the performance of a model; in a computational context such an approach is quite attractive because data could be generated without human intervention, and could even lead to better models with fewer fine-scale computations.

We expect this method to be fully extensible to 2D or 3D geometries, but this would need to be verified. This would require methods such as Convolutional Neural Networks that work well for 2D and 3D systems. In addition, more data management will be required to assemble the many fine-scale and coarse-scale simulations as a training database for the ML approach and to apply the ML model within an LBM simulation. Exploring these tasks is left to future research.

To conclude, we have demonstrated that an ML upscaler can be used to account for discrete molecular effects in larger scale continuum models. This is accomplished by training ML emulators for the fine- and coarse-scale models, and training the ML upscaler to find a mapping between the input spaces of the fine- and coarse-scale models such that their output spaces agree. While we have demonstrated this concept on a simple nanoconfinement example, it is a general framework for scale-bridging that takes advantage of recent advances in ML.

## Methods

### Molecular dynamics simulations

We performed Molecular Dynamics (MD) simulations of methane in a channel pore under equilibrium conditions. We simulated different conditions to construct a robust training data set for our machine learning framework. Given that our main objective is to develop a scale-bridging machine-learning-based workflow, we assumed that pore walls are composed of frozen methane molecules for the sake of simplicity. The simulation domain comprises bulk methane molecules bounded in the z-direction by pore walls as shown in Fig. 1, left side. The x and y dimensions of the simulation box are 42.5 nm and 4.25 nm, similar to the previous work by Li. ^55^. We generated 1000 simulations varying the pore width from 3 to 13 nm, the bulk density from 3 to $250$ kg/m^3^ and the temperature from 298 to 398 K; randomly choosing the values for pore width, bulk density, and temperature for each simulation.

We performed all the simulations using LAMMPS molecular dynamics package ^56^. We use the “fix setforce” command in LAMMPS so that the wall particles do not move during the course of the simulation. Our simulation started with an energy minimization stage to avoid the overlap of molecules, then an equilibration for 0.5 ns and sampling phase for 0.5 ns, both under under NVT (canonical) ensemble. Periodic boundary conditions are employed in the x and y directions. We use a time step of 1 femto-second and a Nosé-Hoover thermostat^57^, with a temperature damping parameter of 10 time units. During the sampling phase, we collected the density profile along the z-direction using a bin size of 0.1 nm. We used a pairwise-additive potential, TraPPE-UA^58^, force field to describe the bulk density from interactions between the methane molecules. The TraPPE force field was developed to describe the bulk properties of the adsorbed gases and has a high degree of accuracy in the prediction of properties at different state points^59^.

### Lattice Boltzmann simulations

#### A brief introduction of the Lattice Boltzmann method

The lattice Boltzmann method^60, 61^ (LBM) is among the most popular direct numerical simulation methods to study complex flow in porous media, thanks to its ability to efficiently implement boundary conditions for complex geometries and to account for interfacial dynamics between different fluids. Furthermore, the LBM is well suited for modern manycore processors/co-processors, such as GPUs (Graphics Processing Units), which greatly boost the computing power but also require a higher degree of explicit parallelism. A highly-optimized LBM code is critical to efficiently provide large number of LBM simulation data to ML training process and also to simulate real rock sample with representative elementary volume (REV). In this work, we implemented the LBM adsorption model on a existing in-house developed high-performance LBM code, ‘MF-LBM’, which is able to simulate complex flow in large 3D complex geometries using manycore processors/co-processors^62, 63^.

The primitive variables in the LBM are the particle distribution functions (PDFs), $f_{i}$, where *i* represents the *i*th lattice direction ***e***. The popular D3Q19 lattice model^64^ is employed in this work. The evolution equations of the PDFs are as follow,

1 $$f_{i} (\user2{x} + \user2{e}_{i} \delta t,t + \delta t) = f_{i} (\user2{x},t) - \frac{{f_{i} (\user2{x},t) - f_{i}^{{eq}} (\user2{x},t)}}{\tau } + F_{i} \delta t,$$

where the LBM single-relaxation-time (SRT) collision model^64, 65^ is employed. Here $f_{i}^{eq}$ are the equilibrium PDFs, $F_i$ represents a general forcing term and the relaxation rate $\tau$ is related to fluid viscosity. For more detailed description of the general LBM, readers may refer to Chen and Doolean^60^.

#### The Shan–Chen type Lattice Boltzmann model

To model methane adsorption in nanopores in LBM simulations, the Shan-Chen single-component-multiphase LBM^66^ is utilized, which generates non-local interactions between fluid-fluid particles and fluid-solid particles. The interactive force between fluid particles can be written as

2 $$\user2{F}(\user2{x},t) = - g\psi (\user2{x},t)\sum\limits_{{i = 1}}^{{18}} {\Omega _{i} } \psi (\user2{x} + \user2{e}_{i} \delta t,t)\user2{e}_{i},$$

where *g* is a parameter that controls the interaction strength, $\psi$ is a function of the local fluid density ***ρ***(***x***) and $\Omega _i$ is the weight factor of ***e***_*i*_ direction in the D3Q19 lattice. The fluid-solid interactive force can be formed in the same way as shown in Eq. (2), by using the fictitious density method. We assign fictitious values of fluid density on the solid nodes, meaning that the values of $\psi$ on the solid nodes are known. Equation (2) is then applied to all the fluid nodes, including the fluid boundary nodes. Thus, ***F***(***x***,*t*) on the fluid boundary nodes can be obtained and show repulsive or attractive depending on the fictitious density assigned on the neighboring solid nodes. This fictitious density method was originally designed to control wettability^67^ on the solid surface, but can also be used to control the adsorption in the present work.

The interactive force obtained in Eq. (2) is incorporated into Eq. (1) using Guo’s forcing scheme^68^, which reduces the discrete lattice effects^69^. The form of $\psi$ in Eq. (2) determines the equation of state^70^. The Peng-Robinson (P-R) equation of state (EOS) has been introduced into the LBM via the following form of $\psi$^70^,

3 $$\begin{aligned} \psi =\sqrt{\frac{2(\frac{\rho RT}{1-b\rho } - \frac{a\alpha (T)\rho ^2}{1+2b\rho -b^2\rho ^2}-c^2_s\rho )}{c^2_s g}}, \end{aligned}$$

where $a=0.45724R^2T^2_c /p_c$; $b=0.00778RT_c /p_c$; *R* is the universal gas constant; $c_s$ is the speed of sound, $T_c$ and $p_c$ are the critical temperature and critical pressure of the gas, respectively; $\alpha (T)=[ 1 + (0.37464+1.54226\omega -0.26992\omega ^2) \times (1-\sqrt{T/T_c})) ]^2$; $\omega =0.011$ is the acentric factor for methane; *T* is the temperature. Following the work of Yuan and Schaefer^70^, we set $a=2/49$, $b=2/21$ and $R=1$ in the simulations. The Shan-Chen multiphase model incorporated with P-R EOS shows significant improvement over the original model in terms of spurious currents, temperature ranges, and density ratio^70^.

From Eq. (3) we can see that *g*, the parameter to control the interaction strength in the original Shan-Chen model, is canceled out. Therefore, the interaction strength is now controlled by *T* and $\rho$.

#### Modeling adsorption in Lattice Boltzmann simulations

As shown in Eq. (2), there are net forces on the fluid boundary nodes if the term $\sum\nolimits_{{i = 1}}^{{18}} {\Omega _{i} \psi (\user2{x} + \user2{e}_{i} \delta t,t){\user2{e}}_{i} }$, which approximates the gradient of $\psi$, does not equal 0 on fluid boundary nodes. Therefore, by tuning the fictitious density on the solid boundary nodes, one can alter the gradient of $\psi$ on the fluid boundary nodes and therefore alter the net interactive forces on the fluid boundary nodes. We define an adsorption parameter,

4 $$\begin{aligned} a'=\frac{\rho _s}{\rho _{total}}, \end{aligned}$$

where $\rho _s$ is the fictitious density on the solid nodes and $\rho _{total}$ is the total density of the fluid. In such case, there are net interactive forces towards the solid nodes on the fluid boundary nodes when $a'>1$, which attract fluid particles to the solid surfaces. This models adsoprtion in the LBM simulation.

#### Matching the density profile in the adsorption layer

The gas density profiles near the solid surface are affected by the adsorption coefficient, the interaction strength in Eq. (2), the choice of EOS, and the forcing scheme that incorporates the interactive forces into the LBM equations. Once the EOS and forcing scheme are chosen, the density profile is determined by the adsorption coefficient $a'$ and the interaction strength which is controlled by *T* and $\rho$ when using the P-R EOS. However, to the best of our knowledge, there are no physics-based LBM that are able to describe the complex fluid-solid interactions inside the adsorption layer.

As described in section “Molecular dynamics simulations”, MD simulations can provide the gas density profile inside the adsorption layer. Therefore, one can tune the parameters, $a'$, *T* and $\rho$, in the LBM simulation to form desired density profile inside the adsorption layer that matches best with the MD result. For convenience, we replace the tuning parameters *T* and $\rho$ with $T_r$ and ${\rho _r}_{total}$ respectively, where $T_r=T/T_c$ is the reduced temperature and ${\rho _r}_{total}={\rho }_{total}/\rho _c$ is the reduced total density. Here $T_r$ and ${\rho _r}_{total}$ are both numerical tuning parameters which are decoupled from the physical temperature and density, and the EOS incorporated in the LBM is only used to provide fluid-solid interactions near solid surface. This is justified by the following two facts:

1. The temperature variation in the simulation length scale of this work (nanoscale) is negligible. Thus, the flow can be considered as isothermal flow. Tuning $T_r$ in the simulation mostly affects the density profiles inside the adsorption layer.
2. The inertial effects in the simulation length scale of this work (nanoscale) is negligible. Thus, one can re-scale the mass unit conversion between the lattice space and physical space.

The above method is in contrast to a previous work^35^, where the physical $T_r$ and ${\rho _r}_{total}$ are used in the LBM simulation. We found that density profiles inside the adsorption layer of the LBM simulations will be also affected by grid resolution and the forcing scheme used in the Shan-Chen model. Only tuning the adsorption parameter $a'$ is not sufficient to obtain good match for a variety of channel widths and physical conditions. Thus, we decoupled $T_r$ and ${\rho _r}_{total}$ in the simulations from the physical ones and used them as tuning parameters. With fast and accurate LBM emulator trained from MD data, as shown in Fig. 4, tuning $a'$, $T_r$, and ${\rho _r}_{total}$ in the LBM simulation simultaneously for different channel widths under certain constraints to match the MD density profiles becomes possible.

### Machine learning workflow

Our machine learning workflow proceeds in three steps: (1) Train an MD-emulator model that maps pore properties to reproduce profiles from MD. (2) Train an LBM-emulator to reproduce profiles from LBM, using an extended parameter set to cover possible adsorption conditions, and (3) Train an upscaler model which maps the MD inputs to effective values of LBM inputs which produce the same profile as estimated from the MD-emulator. We implemented the following methods using PyTorch ^43^.

The notion of two profiles being the same is evaluated using a cost function that compares the mean-squared differences between profiles, scaled by the overall density. We denote distances between an emulated profile $\rho _A(x)$ and a target density $\rho _B(x)$ using a cost function $\mathcal {L}_{\text {emulator}}$:

5 $$\begin{aligned} \mathcal {L}_{\text {emulator}}(\rho _A(x),\rho _B(x)) = \frac{1}{n_i}\sum _i \left( \rho _A(x_i) - \rho _B(x_i)\right) ^2, \end{aligned}$$

where $n_i$ is the number of samples in the profile, and $x_i$ are the sample points.

#### Emulators

The MD-emulator is based on a fully-connected network conditioned on the position within the profile, that is, the predicted profile $\hat{\rho }(x)$ is computed using inputs *x*, *w*, $\rho _0$, and *T* as a function $\hat{\rho }(x) = \rho _0 f(x,w,\rho _0,T)$, where *f* is a neural network. The network output across the profile is normalized to produce a profile density; the output density $\hat{\rho }(x)$ is constrained to obey $\sum _i dx \hat{\rho }(x_i) = \rho _0$. The input positions *x* are transformed via $x \rightarrow \tilde{x} = (2x/w - 1)^2$; this ensures that $\tilde{x}$ is of order 1 (normalizing the feature), as well as ensuring that emulated profiles are symmetric. Put another way, the profile as modeled is a partially applied function $\hat{\rho }(x)|_{w,\rho _0,T}$ that is mathematically equivalent to a Convolutional Neural Network using $1\times 1$ kernels with a single system-wide layer that normalizes the profile to ensure mass conservation; in that sense, our model utilizes parameter-sharing across the pore geometry in the same way that a CNN does.

Our choice to rely on point-wise networks rather than Convolutional Neural Networks is due to the simplifactions that arise for any 1D constant pore geometry (such as the slab analyzed here, or a cylindrical pore geometry): the profile can be treated as a function of 4 variables, rather than a function of $3+w$ variables associated with 3 thermodynamic variables and *w* profile bins. In a CNN-based formulation, the raw data associated with a 1D representation of the pore geometry would be nearly trivial, as it is constant across the pore and constant in the walls. This plays against the advantages of CNNs, which are designed to process local correlations, and would need to either (1) generate the spatial characteristics of the profile using a very large total receptive field size, or (2) explicitly take as input long-range spatial information such as $\tilde{x}$. However, to treat 2D or 3D geometries with more complex spatial structure of the pore walls, a CNN could be employed to represent the geometry of the pore, which is advantageous over parameterizing 2D or 3D functions using purely local variables; said parameterization could easily be accomplished for specific geometries (e.g. ellipses or rectangular cross-sections), but generic irregular shapes would not admit a low-dimensional parameterization.

Futhermore, implementing our model as a point-wise full-connected model is also computationally advantageous over a CNN formulation because it avoids some of the difficulties associated with treating variable-sized inputs. Treating variable-sized inputs requires either (1) padding all data to the size of the largest sample in the batch, resulted in wasted computation associated with the profile inside a wall, which vanishes, or (2), calling the network only on batch sizes of 1, which limits the computational throughput of training by restricting parallelism. In the case of a 2D or 3D geometry, these concerns would be mitigated as a result of the larger number of per-example input and output bins.

Networks consist of $n_{layers}=3$ (i.e. two hidden layers and one output layer) of $n_{neurons}=30$ each using the softplus activation function $\text {softplus}(x) = \log (1+e^x)$. The use of this smooth activation function ensures that emulated profiles are themselves smooth. Each network contains approximately 1,100 parameters in total. Networks are trained over $n_{epochs}=1{,}000$ using the Adam optimizer^71^. $10\%$ of the data is held out for testing, and another $10\%$ is held out for early stopping and validation. The emulator networks are trained in batches of size $n_{batch}=20$ under the cost function $\mathcal {L}_{\text {emulator}}(\hat{\rho }_{\text {MD}}(x),\rho _{\text {MD}}(x))$.

The LBM dataset of 8,074 calculations was obtained by filtering data obtained from a set of 10,000 calculations. 8,245 of the calculations converged within the number of steps prescribed. The data was filtered to remove simulations for which a unique profile is ill-defined due to the appearance of complete condensation, usually at low temperatures and/or large adsorption coefficients. The filtering process removed simulations for which the observed profile was asymmetric as well as simulations for which the bulk density was greater than the density near the wall. The LBM-emulator is trained in the same fashion, except that it has inputs $w,\rho _0',T',a'$. The densities $\rho '$ and temperatures $T'$ are expressed in lattice units. The adsorption $a'$ is dimensionless.

#### Upscaler

The goal of the upscaling network is to match emulated MD profiles to emulated LBM profiles by learning a mapping between the MD input space and the LBM input space, producing LBM predictions $\hat{\rho _0}', \hat{T'}, \hat{a'}$ from MD variables $w,\rho _0,T$. To constrain the upscaler as a density-conserving predictor, the upscaled density $\hat{\rho }'$ is learned using a linear factor $\hat{\rho }_0' = \beta _\rho \rho _0$ for a single scalar parameter $\beta _\rho$; the upscaler fixes the density scale matching between MD and LBM; this can likewise be rephrased as a global modification to the LBM critical pressure. The other parameters, $\hat{T}'$ and $\hat{a}'$, are generated by a fully connected multitask neural network. The archtecture is same as the emulators, except that the predictions are made using a linear layer (no activation function), the position is not an input to the upscaler, and there is no profile-wise normalization layer. Like the emulators, the upscaler contains approximately 1100 parameters.

Initial explorations used a similar cost function to the emulators, attempting to match the full density profile. However, it was soon discovered that this task is not possible within the framework of LBM examined; it cannot reproduce, for example, the multi-layer structure observed in the MD profiles. As such, the cost function focuses on the primary observable effect of adsorption: The excess density $\rho _{\text {excess}}$, with a small regularization term encouraging the upscaler to keep the MD and upscaled profiles close:

6 $$\begin{aligned} \mathcal {L}_{\text {upscaler}} = \left( (\rho _{\text {excess,upscaled}}-\rho _{\text {excess,MD}} )^2+ 0.01 * \mathcal {L}_{\text {emulator}}(\hat{\rho }_{\text {upscaled}},\hat{\rho }_{\text {MD}}) \right) / \rho _{0}. \end{aligned}$$

One advantage of this approach is that the set of input parameters for MD needs not be explicitly matched to LBM inputs; the upscaler itself solves this problem implicitly, as both emulators are defined over the space of inputs in the data collected. As such, we train the upscaler over a large dataset of emulated MD profiles that cover the space of both the MD and LBM simulations. This consists of 10,000 calculations with widths between 2.5 and 12.5 nm, densitites between 2.5 kg/m^3^ and 250 kg/m^3^, and temperatures between 300 and 400 K. The upscaler is trained for 100 epochs.
